# The Pathological Phenotypes of Human TDP-43 Transgenic Mouse Models Are Independent of Downregulation of Mouse Tdp-43

**DOI:** 10.1371/journal.pone.0069864

**Published:** 2013-07-26

**Authors:** Ya-Fei Xu, Mercedes Prudencio, Jaime M. Hubbard, Jimei Tong, Ena C. Whitelaw, Karen Jansen-West, Caroline Stetler, Xiangkun Cao, John Song, Yong-Jie Zhang

**Affiliations:** Department of Neuroscience, Mayo Clinic Jacksonville, Jacksonville, Florida, United States of America; Louisiana State University Health Sciences Center, United States of America

## Abstract

Tar DNA binding protein 43 (TDP-43) is the major component of pathological deposits in frontotemporal lobar degeneration with TDP-43 inclusions (FTLD-TDP) and in amyotrophic lateral sclerosis (ALS). It has been reported that TDP-43 transgenic mouse models expressing human TDP-43 wild-type or ALS-associated mutations recapitulate certain ALS and FTLD pathological phenotypes. Of note, expression of human TDP-43 (hTDP-43) reduces the levels of mouse Tdp-43 (mTdp-43). However, it remained unclear whether the mechanisms through which TDP-43 induces ALS or FTLD-like pathologies resulted from a reduction in mTdp-43, an increase in hTDP-43, or a combination of both. In elucidating the role of mTdp-43 and hTDP-43 in hTDP-43 transgenic mice, we observed that reduction of mTdp-43 in non-transgenic mice by intraventricular brain injection of AAV1-sh*Tardbp* leads to a dramatic increase in the levels of splicing variants of mouse sortilin 1 and translin. However, the levels of these two abnormal splicing variants are not increased in hTDP-43 transgenic mice despite significant downregulation of mTdp-43 in these mice. Moreover, further downregulation of mTdp-43 in hTDP-43 hemizygous mice, which are asymptomatic, to the levels equivalent to that of mTdp-43 in hTDP-43 homozygous mice does not induce the pathological phenotypes observed in the homozygous mice. Lastly, the number of dendritic spines and the RNA levels of TDP-43 RNA targets critical for synapse formation and function are significantly decreased in symptomatic homozygous mice. Together, our findings indicate that mTdp-43 downregulation does not lead to a loss of function mechanism or account for the pathological phenotypes observed in hTDP-43 homozygous mice because hTDP-43 compensates for the reduction, and associated functions of mTdp-43. Rather, expression of hTDP-43 beyond a certain threshold leads to abnormal metabolism of TDP-43 RNA targets critical for neuronal structure and function, which might be responsible for the ALS or FTLD-like pathologies observed in homozygous hTDP-43 transgenic mice.

## Introduction

Tar DNA-binding protein 43 (TDP-43) is the principal component of ubiquitinated inclusions in frontotemporal lobar degeneration with TDP-43-positive inclusions (FTLD-TDP) and amyotrophic lateral sclerosis (ALS) [1], [2]. Mutations in *TARDBP*, the gene encoding TDP-43, are associated with sporadic and familial ALS, indicating that TDP-43 can directly cause neurodegeneration [3], [4], [5], [6], [7]. Normally, TDP-43 localizes to the nucleus; however, a substantial loss of nuclear TDP-43 is observed in neurons bearing aberrant cytoplasmic TDP-43 inclusions in disease [1], [2]. The mechanisms through which TDP-43 proteins mediate neurodegeneration remains unclear, though it is believed to be attributable to either a toxic gain of function, a loss of function, or a combination of both.

Previous studies have explored the TDP-43 loss of function hypothesis *in vitro* and *in vivo* [8], [9], [10], [11]. Wu and colleagues disrupted the expression of mouse Tdp-43 (mTdp-43) *in vivo* and observed that mice developed early embryonic lethality, suggesting an important role for TDP-43 in development [8]. Conditional downregulation of mTdp-43 in mouse spinal cord led to the development of ALS-like phenotypes supporting the hypothesis that loss of TDP-43 function is a major cause of neurodegeneration in ALS [9]. Several other studies demonstrated the potential contribution of TDP-43 deficiency to disease pathogenesis [10], [12], while our group and others have demonstrated that overexpression of the human TDP-43 (hTDP-43) protein, either wild-type (hTDP-43_WT_) or mutant hTDP-43, leads to pathological phenotypes consistent with certain TDP-43 proteinopathies. These phenotypes may include some of the following: increased ubiquitination, truncation, aggregation and phosphorylation of TDP-43, cytoplasmic TDP-43 inclusions, neuronal degeneration, motor dysfunction, learning and memory deficits, and mitochondrial abnormalities [13], [14], [15], [16], [17], [18], [19]. Moreover, we [14], [15] and others [12] have observed that expression of hTDP-43 protein in transgenic mice decreases the mRNA levels of endogenous mouse *Tardbp*. Additionally, recent studies demonstrate that TDP-43 autoregulates itself to decrease its expression [20], suggesting that hTDP-43 may thus decrease mouse *Tardbp* levels through this mechanism. However, it remains unclear whether the reduction in mTdp-43 (loss of function) or the overexpression of hTDP-43 (gain of function), or combined events are responsible for the ALS or FTLD-like pathologies observed in hTDP-43 transgenic mice.

To address the aforementioned question, we first generated an antibody that specifically detects mTdp-43 protein, and confirmed that downregulation of mouse *Tardbp* mRNA observed in our hTDP-43 transgenic mice results in significant reduction of mTdp-43 protein. We found that this reduction in mTdp-43 does not increase the levels of splicing variants known to be inhibited by TDP-43. In particular, the levels of mouse Ex17b-containing sortilin 1 (*mSort1+Ex17b*) and Ex5-deleted translin (*mTsn_*Δ*Ex5*) mRNAs are significantly upregulated when mTdp-43 protein is reduced in non-transgenic (NT) mice but not in hTDP-43 mice, suggesting that hTDP-43 has similar biological functions to that of mTdp-43 *in vivo*. Moreover, a further reduction of mTdp-43 in asymptomatic hemizygous hTDP-43 (hTDP-43_hemi_) mice to the levels equivalent to that of symptomatic homozygous hTDP-43 (hTDP-43_homo_) mice did not lead to the development of pathological phenotypes indicating that downregulation of mTdp-43 is not sufficient to induce the pathological phenotypes observed in hTDP-43_homo_ mice. Finally, we provide evidence that high expression levels of hTDP-43 beyond a certain threshold lead to alterations in TDP-43 RNA substrates critical for maintaining neuronal structure and function and this, in turn, might cause neuronal dysfunction and the development of pathological phenotypes.

## Results

### The Protein Levels of Mouse Tdp-43 are Reduced in Human TDP-43 Transgenic Mice

**Figure 1:**
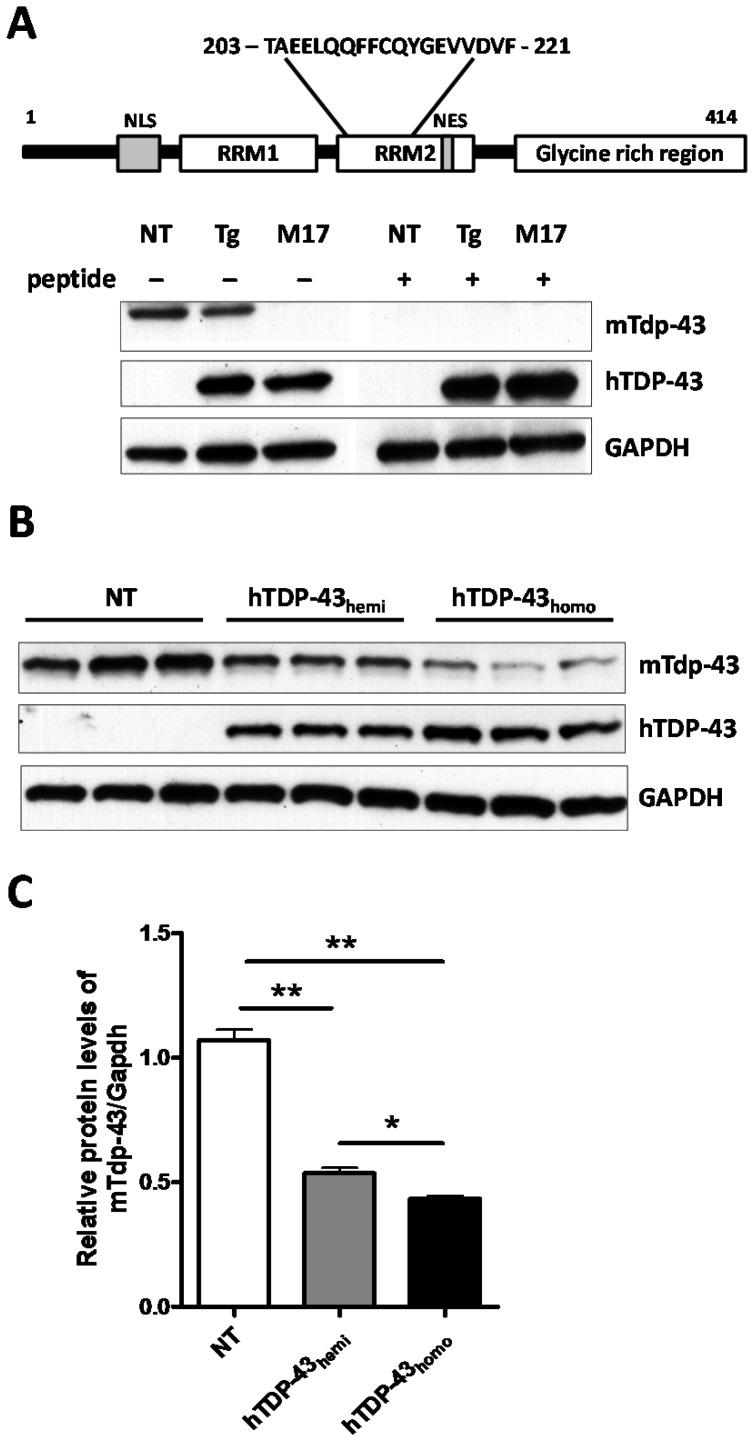
The protein levels of mouse Tdp-43 are reduced in human TDP-43 transgenic mice. (**A**) Generation and characterization of an antibody that specifically detects mouse Tdp-43 (mTdp-43 antibody). Schematic representation of mouse TDP-43 protein and the amino acid residues used as immunizing peptide. Western blot analyses of lysates from mouse brain of non-transgenic (NT) and human TDP-43 transgenic (Tg) mouse, and human M17 neuroblastoma cells. Before proceeding with the staining protocol, mTdp-43 antibody is neutralized with immunizing peptide. The neutralized antibody is then used side-by-side as a control for the antibody alone. Our results showed that our mTdp-43 antibody can specifically recognize mTdp-43 in both NT and hTDP-43 Tg mice brain, but cannot recognize human TDP-43 in human sample. Human TDP-43 antibody (hTDP-43) was used to confirm the expression of human TDP-43 in samples. (**B**) Compared to NT mice, significant reduction of mTdp-43 protein was observed in both TDP-43 hemizygous and homozygous mice brain. Data shown are the means ± SEM of 5 mice per group; *p<0.05, **p<0.001, as assessed by one-way ANOVA with Tukey’s posthoc analysis.

Recently, we and others have reported that the mRNA levels of mTdp-43 are significantly decreased in hTDP-43 transgenic mice [12], [14], [15]; however, it had remained unclear whether levels of mTdp-43 protein are similarly decreased in hTDP-43 transgenic mice. As such, we generated a novel antibody that specifically recognizes the mTdp-43 protein, since both mTdp-43 and hTDP-43 proteins are indistinguishable in size by Western blot techniques and there are no commercially available TDP-43 antibodies that recognize the mouse but not the human TDP-43 protein. In doing so, we immunized rabbits with a synthetic peptide corresponding to residues 203–221 of mTdp-43 protein (Fig. 1A). The resulting sera were then affinity purified and the specificity of the resulting mTdp-43 antibody was characterized by Western blot. We observed that our mTdp-43 antibody recognizes mTdp-43 protein from mouse brain lysates of non-transgenic (NT) and hTDP-43 mice, but not the hTDP-43 protein present in human neuroblastoma M17 cells (Fig. 1A). Moreover, incubation of the peptide used for the generation of this antibody with mouse brain lysates blocked the detection of mTdp-43 protein but did not interfere with the ability of human specific TDP-43 antibodies to detect hTDP-43 protein in hTDP-43 mice (Fig. 1A), further verifying the specificity of the antibody. By using this new antibody, we examined the protein levels of mTdp-43 in NT, hTDP-43_hemi_ and hTDP-43_homo_ transgenic mice. The levels of mTdp-43 protein in hTDP-43_hemi_ and hTDP-43_homo_ mice respectively decreased to ∼55% and 45% of NT mice levels (Fig. 1B and C), suggesting that hTDP-43 overexpression downregulates the levels of mTdp-43, but that the downregulation is modest once hTDP-43 expression levels exceed a certain threshold. We also observed downregulation of mouse *Tardbp* mRNA and mTdp-43 protein levels in both hemizyogous and homozygous hTDP-43_M337V_ mice (data not shown). Of importance, TDP-43_M337V_ and TDP-43_WT_ display similar biological activity in autoregulating the levels of TDP-43 in vivo, suggesting the ALS-associated M337V mutation does not lead to loss of function.

### Expression of hTDP-43 Rescues the Abnormal Splicing Variants Induced by mTdp-43 Downregulation in Human TDP-43 Transgenic Mice

**Figure 2:**
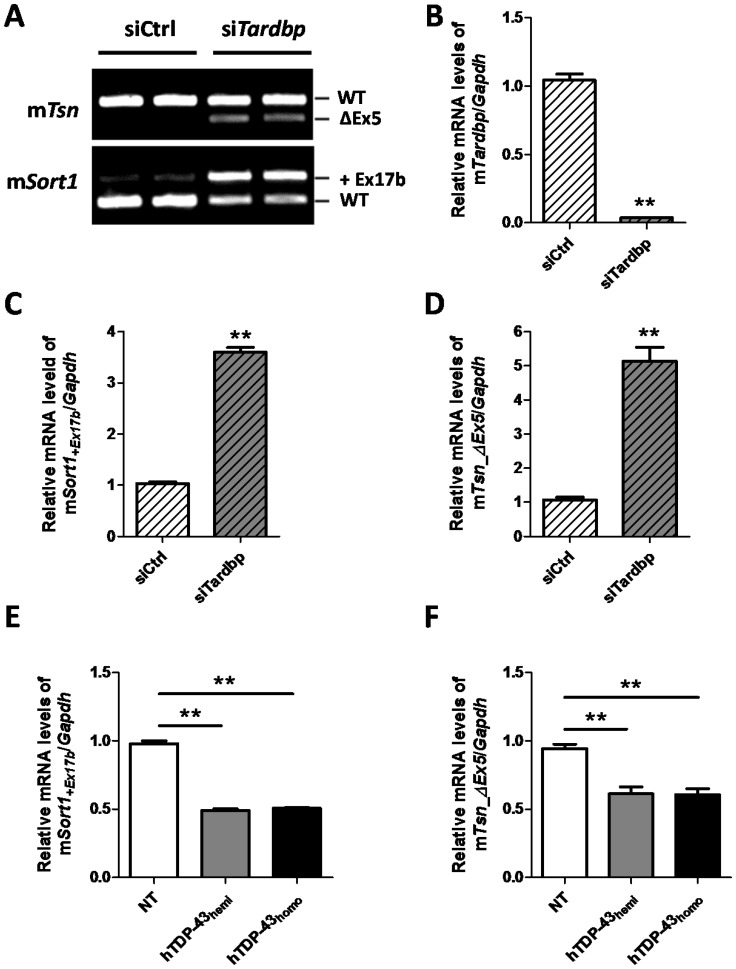
Expression of hTDP-43 rescues the abnormal splicing variants induced by mTdp-43 downregulation in human TDP-43 transgenic mice. (**A–D**) Knockdown of mTdp-43 induces abnormal splicing of TDP-43 RNA targets mouse *sortilin 1* (*Sort1*) and *translin* (*Tsn*) in N2a cells. (**A**) Knockdown of mTdp-43 in N2a cell line using small inference RNA (siRNA) leads to generation of abnormal splicing variants *mSort1+Ex17b* and *mTsn_*Δ*Ex5*, indicating loss of mTdp-43 function. (**B–D**) Quantitative PCR (q-PCR) is used to quantify the mRNA levels of mTdp-43, *mSort1+Ex17b* and *mTsn_*Δ*Ex5*. The levels of *mSort1+Ex17b* and *mTsn* are significantly increased when Tdp-43 is depleted. (**E–F**) The mRNA levels of *mSort1+Ex17b* (**E**) and *mTsn_*Δ*Ex5* (**F**) are not increased in hTDP-43 hemizygous and homozygous mice brain. In contrast, the levels of these two abnormal splicing variants are reduced dramatically in hTDP-43 transgenic mice. Data shown are the means ± SEM of 5 mice per group; **p<0.001, as assessed by one-way ANOVA with Tukey’s posthoc analysis.

One of the well characterized functions of TDP-43 is its ability to regulate the splicing of its RNA targets, and loss of mTdp-43 has been shown to result in the generation of abnormal splicing variants of some of these targets in cells and *in vivo* [20], [21]. Since the levels of mTdp-43 protein are significantly decreased in our hTDP-43 transgenic mice (Fig. 1), we sought to determine whether there is loss of mTdp-43 function in these mice by evaluating the ability of mTdp-43 to regulate splicing of two of its RNA targets: *sortilin 1* (*Sort1*) [20], [21] and *translin* (*Tsn*) [20]. Consistent with previous reports [20], [21], downregulation of mTdp-43 in mouse N2a neuroblastoma cells by small inference RNA (siRNA) significantly increased the levels of the abnormal splicing variants: *mSort1+Ex17b* (sortilin 1 including exon 17b) and *mTsn_*Δ*Ex5* (translin with exon 5 deletion) (si*Tardbp*, Fig. 2A). To further quantify this data, we designed primers for quantitative real time PCR (qRT-PCR) to specifically measure the levels of mouse *Tardbp*, *mSort1+Ex17b* and *mTsn_*Δ*Ex5*. Compared to cells treated with a control siRNA (siCtrl), the levels of *mSort1+Ex17b* and *mTsn_*Δ*Ex5* increased 3.7- and 5.2-fold, respectively, when mTdp-43 levels are reduced (si*Tardbp*, Fig. 2B–D). Then, by using those primers, we measured the levels of *mSort1+Ex17b* and *mTsn_*Δ*Ex5* in hTDP-43 transgenic mice. Surprisingly, while mTdp-43 protein is significantly decreased in hTDP-43 transgenic mice (Fig. 1B–C), the levels of *mSort1+Ex17b* and *mTsn_*Δ*Ex5* splicing variants were not increased compared to NT mice (Fig. 2E and F). Similar results regarding the levels of *mSort1+Ex17b* and *mTsn_*Δ*Ex5* were observed in hTDP-43_M337V_ transgenic mice (Fig. S1D, E).

**Figure 3:**
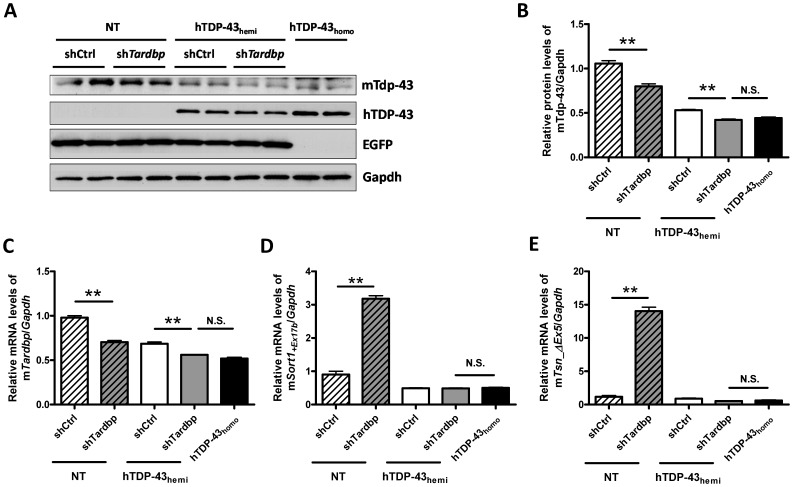
Intraventricular injection of AAV1-sh*Tardbp* in neonatal mouse brain results in loss of TDP-43 function in non-transgenic mice, but not human TDP-43 hemizygouse transgenic mice. (**A–C**) Compared to AAV1-shCtrl, intraventricular injection of AAV1-sh*Tardbp* reduces the levels of mTdp-43 protein (**A** and **B**) and mRNA (**C**) in brains of both non-transgenic (NT) and hTDP-43_WT_ hemizygous mice. Of note, co-expression of EGFP with shRNA confirmed the transduction efficiency is comparable among treated groups (**A**). Moreover, the mTdp-43 protein and RNA levels between AAV1-sh*Tardbp* injected hemizygous mice and homozygous mice are not significantly different. (**D–E**) Injection of AAV1-sh*Tardbp* significantly increases the mRNA level of *mSort1+Ex17b* (**D**) and *mTsn_*Δ*Ex5* (**E**) in the brain of NT mice, but not in hTDP-43_WT_ hemizygous mice. There is no significant difference of *mSort1+Ex17b* and *mTsn_*Δ*Ex5* RNA levels between AAV1-sh*Tardbp* injected hemizygous mice and homozygous mice. Data shown are the means ± SEM of 3–5 mice per group; **p<0.001, N.S. no significance, as assessed by One-way ANOVA with Tukey’s posthoc analysis.

To determine whether downregulation of mTdp-43 *in vivo* can increase the levels of *mSort1+Ex17b* and *mTsn_*Δ*Ex5* splicing variants, we performed intraventricular brain injections of adeno-associated virus type 1 (AAV1)-shRNA targeting mTdp-43 in neonatal mice expressing only mTdp-43 (NT mice) or mTdp-43 and hTDP-43 (hTDP-43_hemi_ mice). Note that efficient downregulation of mTdp-43 protein and RNA was achieved in both NT and hTDP-43_hemi_ transgenic mice (Fig. 3A–C). Co-expression of EGFP with shRNA confirmed the transduction efficiency is comparable among treated groups (Fig. 3A). Moreover, downregulation of mTdp-43 in NT mice dramatically increased the levels of *mSort1+Ex17b* and *mTsn_*Δ*Ex5* (Fig. 3D, E). However, compared to hTDP-43_hemi_ mice transduced with a control shRNA (shCtrl), the levels of *mSort1+Ex17b* and *mTsn_*Δ*Ex5* were not increased after further reduction of mTdp-43 in sh*Tardbp*-treated hTDP-43_hemi_ mice. Similar results were observed in hTDP-43_M337V_ mice (Fig. S1). These data suggest that human TDP-43 protein expressed in these mice is inhibiting the formation of the *mSort1+Ex17b* and *mTsn_*Δ*Ex5* mouse splicing isoforms.

### A Reduction in the Levels of Mouse Tdp-43 does not Account for the Pathological Phenotypes Observed in Human TDP-43 Homozygous Transgenic Mice

**Figure 4:**
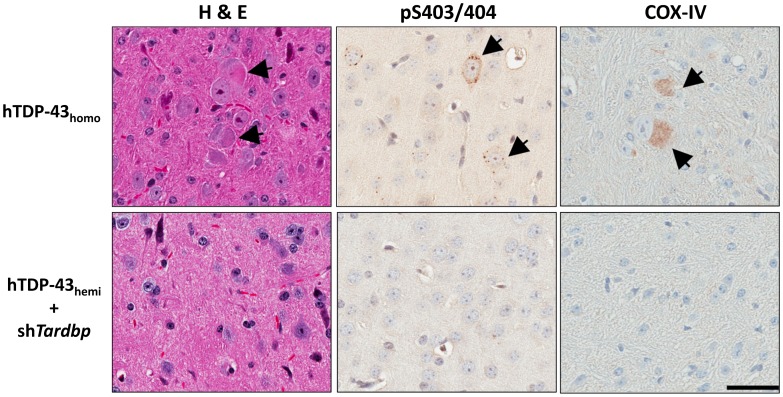
Further downregulation of mTdp-43 in the brain of hTDP-43 hemizygous mice do not induce pathologies observed in hTDP-43 homozygous mice. (**A–B**) Hematoxylin and eosin staining (H&E) shows that eosinophilic aggregates in neurons from the brain sections of 1 month old hTDP-43 homozygous mice (**A**) are not observed in AAV1-sh*Tardbp* injected hTDP-43 hemizygous mice (**B**). (**C–D**) Phosphorylated TDP-43 aggregates are only observed in the neurons of the brain sections of 1 month old hTDP-43 homozygous mice (**C**), but not in AAV1-sh*Tardbp* injected hTDP-43 hemizygous mice (**D**). (**E–F**) COX-IV immunoreactivity illustrates densely stained mitochondria aggregates in neurons from the brain sections of 1 month old hTDP-43 homozygous mice (**E**), but not in AAV1-sh*Tardbp* injected hTDP-43 hemizygous mice (**F**). Scale bars: 100 µm.

We have previously reported that hTDP-43_homo_ mice develop certain ALS or FTLD-like pathologies; however, hTDP-43_hemi_ mice are phenotypically indistinguishable from NT mice [14], [15]. Compared to hTDP-43_hemi_, hTDP-43_homo_ mice have lower mTdp-43 protein levels, but higher hTDP-43 protein levels (Fig. 1). To investigate whether the lower levels of mTdp-43 in hTDP-43_homo_ mice are responsible for the phenotypic differences between hTDP-43_homo_ and hTDP-43_hemi_ mice, we treated hTDP-43_hemi_ mice with AAV-sh*Tardbp* and compared their phenotype to that of hTDP-43_homo_ mice (see Fig. 3A–C). Since hTDP-43_homo_ mice developed pathological phenotypes at 1 month of age [14], [15], we harvested hTDP-43_hemi_ mice 1 month after administration of AAV-sh*Tardbp*. Compared to shCtrl-treated hTDP-43_hemi_ mice, administration of AAV1-sh*Tardbp* significantly decreased the mRNA and protein levels of mTdp-43 to the levels equivalent to that of hTDP-43_homo_ mice (Fig. 3A–C). At 1 month old, hTDP-43_homo_ mice develop ALS and FTLD-like pathologies such as cytoplasmic eosinophilic aggregates (Fig. 4A), phosphorylated TDP-43 inclusions (Fig. 4B), and abnormal mitochondrial aggregation (Fig. 4C). These pathological features were absent in both AAV1-sh*Tardbp* injected hTDP-43_hemi_ mice (Fig. 4D–F) and NT mice (data not shown). In addition, sh*Tardbp*-treated hTDP-43_hemi_ mice did not develop any motor deficits or early mortality that is characteristic of hTDP-43_homo_ mice (data not shown). Overall our data suggest that a decrease in mTdp-43 protein in hTDP-43 transgenic mice is not responsible for the observed phenotype in these mice. Rather, it does seem that the human TDP-43 may be driving these events.

### High Levels of Human TDP-43 Decrease the RNA Levels of Synaptic Proteins and Reduce the Number of Dendritic Spines *in Vivo*

**Figure 5:**
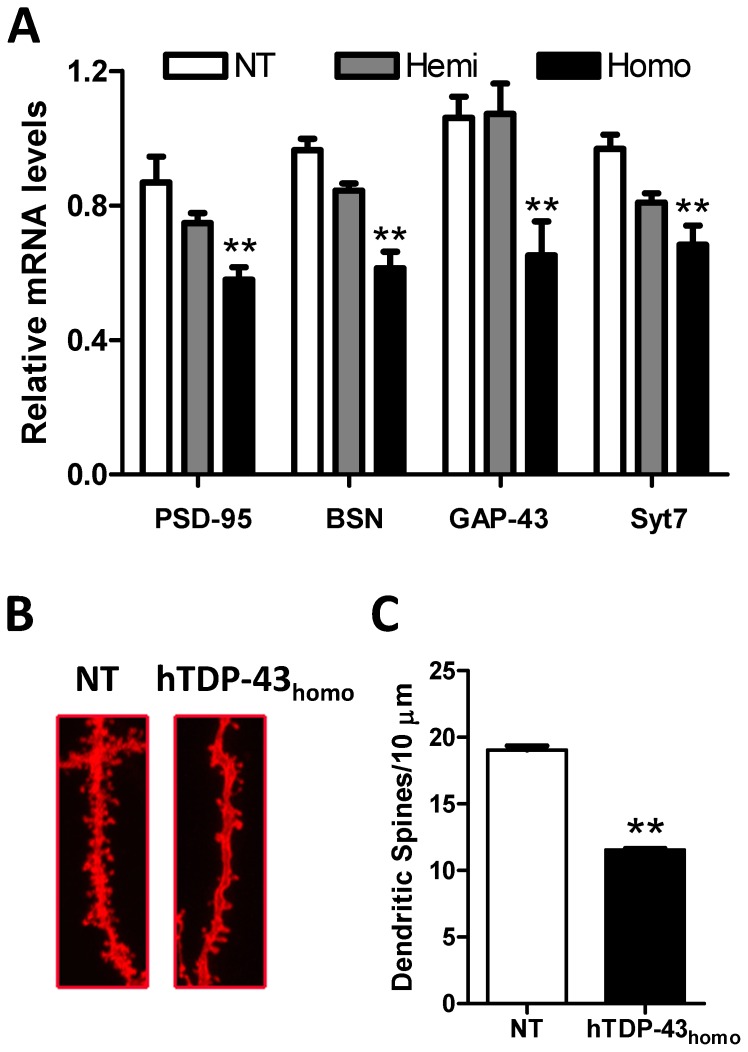
Alternation of RNA levels of synaptic proteins and dendritic spines in hTDP-43_WT_ homozygous mice. (**A**) Compared with non-transgenic (NT) mice, the RNA levels of TDP-43 targets PSD-95, Bsn, GAP-43 and Syt7 are significantly decreased in TDP-43 homozygous mice, but not in TDP-43 hemizygous mice. (**B–C**) The numbers of dendritic spine are dramatically reduced in the brain of TDP-43 hemozygous mice compared to NT mice. Data shown are the means ± SEM of 3–5 mice per group; **p<0.001, as assessed by one-way ANOVA with Tukey’s posthoc analysis or Two-tailed student’s t test to compare measures among 2 groups.

Given our data indicate that hTDP-43 compensates for loss of mTdp-43 in hTDP-43_homo_ mice to a certain point, beyond which the overexpression of hTDP-43 induces toxicity, we sought to investigate whether the highly abundant hTDP-43 protein present in these mice would alter the levels of certain TDP-43 mRNA targets and consequently account for the TDP-43 associated pathologies in hTDP-43_homo_ mice. To this end, we measured the levels of several neuronal TDP-43 RNA targets that are critical for neuronal survival and function [22]. We measured the following RNA targets: bassoon (Bsn), discs large homolog 1 (Dlg1), discs large homolog 2 (Dlg2), discs large homolog 4 (Dlg4, also called Psd-95), segment polarity protein dishevelled homolog (DVL-1), growth associated protein 43 (Gap-43), synaptosomal-associated protein 25 (Snap-25), synaptotagmin 1 (Syt1), and synaptotagmin 7 (Syt7). Among these, the mRNA levels of Psd-95, Bsn, Gap-43, and Syt7 were found to be dramatically reduced in hTDP-43_homo_ mice compared to NT mice and hTDP-43_hemi_ mice (Fig. 5A). Since the proteins encoded by these TDP-43 RNA targets are known to play important roles in synaptic function and formation of dendritic spines [23], [24], we evaluated the number of dendritic spines in hTDP-43_homo_ compared to NT mice. We observed that the number of dendritic spines is significantly reduced in hTDP-43_homo_ mice (Fig. 5B, C). Of note, the RNA levels of these synaptic proteins and the number of dendritic spines are not reduced in AAV1-sh*Tardbp* injected hTDP-43_hemi_ mice (Fig. S2A–C), indicating that expression of hTDP-43 over in excess of a certain level, rather than downregulation of mTdp-43, causes abnormal metabolism of TDP-43 mRNA targets in hTDP-43 transgenic mouse models.

## Discussion

Generation of transgenic mice is a widely used research technique to model human diseases, given transgenic mice recapitulate key pathological features of disease and also serve as valuable tools to explore the role of disease-causing molecules (e.g. TDP-43). More importantly, the models provide opportunities to evaluate potential therapeutic strategies for translational research. For instance, mutations in genes encoding amyloid precursor protein (*APP*), presenilins (*PSEN1* and *PSEN2*) or microtubule associated protein tau (*MAPT*) have been overexpressed in animals to make Alzheimer’s disease (AD) mouse models, which are characterized by the production and accumulation of amyloid-β into plaques or of hyperphosphorylated tau into neurofibrillary tangles [25], [26]. Moreover, mutations in the SOD1 gene have also been used to generate transgenic mouse models, which develop the progressive loss of motor neurons, muscle weakness and atrophy, and eventual death similar to the pathological phenotypes in ALS [27].

In 2006, TDP-43 was identified as the principal protein component of pathological inclusions in FTLD-TDP and ALS [1], [2]. Mutations in the *TARDBP* gene have also been found in sporadic and familiar ALS patients, further supporting that alterations of TDP-43 directly cause ALS [3], [4], [5], [6], [7]. Since then, several transgenic mouse models that either over-express human wild-type TDP-43 or ALS-associated TDP-43 mutations have been generated and characterized [12], [13], [14], [15], [16], [17]. Briefly, these mouse models share very similar pathological phenotypes such as ubiquitin accumulation, TDP-43 fragmentation, astrogliosis, microgliosis, axonal degeneration, neuronal loss, motor function impairment, and shortened lifespan [12], [13], [14], [15], [16], [17], [18], [19]. In some of those models, phosphorylated TDP-43 inclusions are rarely observed in the brain and spinal cord [12], [14], [15], [28].

Given that TDP-43 plays critical roles in RNA metabolism, such as splicing, transport and transcription [29], excessive levels of hTDP-43 in transgenic models might result in the disruption of RNA metabolism. Interestingly, several studies revealed that TDP-43 protein binds to the 3′-UTR of its mRNA, and auto-regulates its levels through combined mechanisms [20], [30], [31]. We, as well as others, have observed that the mRNA levels of mTdp-43 are significantly decreased in hTDP-43 transgenic mice [12], [14], [15], [20]. Furthermore, it has been shown that downregulation of mTdp-43 alters splicing events of multiple mRNAs [20], and that conditional downregulation of mTdp-43 *in vivo* leads to ALS-like phenotypes including kyphosis, motor dysfunction, muscle atrophy, and astrocytosis [9]. These data suggest that the development of disease in hTDP-43 transgenic mice may result from loss of mTdp-43 function; however, whether the overexpression of high levels of hTDP-43 in mice accounts for any of the disease-associated phenotypes remained unknown.

In the present study, we used our hTDP-43 transgenic mouse models to determine whether the disease phenotypes that develop in these mice result from high hTDP-43 protein levels or by the downregulation of endogenous mTdp-43 protein. To help us address this question, we generated an antibody that specifically detects mTdp-43 to measure protein levels *in vivo*. By using this antibody, we observed that mTdp-43 protein is significantly decreased in hTDP-43 mice compared to NT mice, suggesting that hTDP-43 may be regulating mTdp-43 expression through downregulation of mouse *Tardbp* RNA. This idea is consistent with previous findings that elucidated TDP-43′s autoregulation ability [20], [30], [31].

To further determine whether the lower levels of mTdp-43 protein in our hTDP-43 transgenic mice result in loss of mTdp-43 function, we examined the levels of abnormal splicing variants of two of its RNA targets: *Sort1*, which encodes multi-ligand type 1 transmembrane receptor [32], and *Tsn*, encoding a recombinant hotspot DNA-binding protein [33]. When mTdp-43 levels are decreased in NT mice, mRNA levels of m*Sort1* containing exon 17b (99 base pairs of intron 17, *mSort1+Ex17b*) and m*Tsn* lacking exon 5 (m*Tsn_*Δ*Ex5*) increase dramatically. These two opposite splicing patterns upon mTdp-43 downregulation (exon inclusion in m*Sort1* and exon exclusion in m*Tsn*) make our assay more comprehensive. Of note, the levels of *mSort1+Ex17b* and *mTsn_*Δ*Ex5* are not increased in hTDP-43 transgenic mice, despite the fact that these mice present lower levels of mTdp-43 protein than in AAV1-sh*Tardbp* injected NT mice, which do show a significant increase in these aberrant splicing isoforms. Moreover, further reduction of mTdp-43 in hTDP-43_hemi_ mice to the levels equivalent to that of hTDP-43_homo_ mice do not lead to an increase in m*Sort1+Ex17b* nor m*Tsn_ΔEx5* isoforms. As a result, our data indicate that loss of mTdp-43 function in hTDP-43 mice is compensated by the expression of hTDP-43. These results are not surprising since mouse and human TDP-43 proteins are 96% homologous [28]. We have reported that homozygous transgenic mice of both hTDP-43_WT_ and hTDP-43_M337V_ develop phenotypes characteristic of FTLD and ALS diseases. However, hTDP-43_hemi_ transgenic mice are virtually indistinguishable from NT controls [14], [15]. Compared to the hTDP-43_homo_ mice hTDP-43_hemi_ mice have ∼75% hTDP-43 protein or ∼25% less hTDP-43 protein. As such, we hypothesize that expression of hTDP-43 over a certain threshold will disrupt the processing of TDP-43 RNA targets that will consequently cause the neuropathologies observed in hTDP-43_homo_ mice. While we were unable to detect differences in TDP-43 RNA targets known to be altered by mTdp-43 downregulation, we found significant changes in TDP-43 RNA targets that are critical for neuronal function and survival in hTDP-43 mice overexpressing high levels of hTDP-43. In particular, the mRNA levels of synaptic markers Psd-95, Bsn, Gap-43, and Syt7 are significantly decreased in hTDP-43_homo_ mice, but not in hTDP-43_hemi_ mice. Moreover, the number of dendritic spines in the hippocampus of hTDP-43_homo_ mice is also significantly decreased compared to NT. More important, the mRNA levels of the above mentioned targets and the number of dendritic spines are not altered in AAV1-sh*Tardbp* treated hTDP-43_hemi_ mice, excluding the possibility that low levels of mTdp-43 are responsible for the synaptic dysfunction in hTDP-43_homo_ mice. Together, our results suggest that expression of total levels of TDP-43 protein (human and mouse) above 2.5 fold of endogenous mouse Tdp-43 in NT mice might disrupt RNA metabolism of several synaptic targets [14], [15], [17], [19], [34], consequently leading to neuronal dysfunction in mice.

In conclusion, although expression of hTDP-43 reduces the levels of mTdp-43 in hTDP-43 transgenic mice, this reduction does not lead to loss of mTdp-43 function because hTDP-43 compensates for functional losses in mTdp-43. Moreover, expression of hTDP-43 above a certain threshold disrupts RNA metabolism, especially of genes critical for neuronal survival and function, which consequently causes the pathological phenotypes observed in hTDP-43_homo_ mice. In addition, there is no difference between wild-type and M337V hTDP-43 proteins regarding their ability to regulate RNA splicing. Our findings suggest that the current hTDP-43 transgenic mouse models might not be the most suitable tools to investigate loss of mTdp-43 function. Knock-in transgenic mouse models expressing either wild-type hTDP-43 or ALS-associated TDP-43 mutations at similar levels to mTdp-43 should be generated to understand the roles of TDP-43 in disease pathogenesis.

## Materials and Methods

### TDP-43 Transgenic Mice

The hTDP-43 transgenic mice expressing wild-type or M337V mutant hTDP-43 have been previously reported [14], [15]. The intraventricular injection of virus in mouse brain was performed according to a previous report [35]. Briefly, 2 µl of AAV1-shTardbp or AAV1-shControl virus were injected into the lateral ventricles of postnatal day 0 mice. EGFP is co-expressed with ShRNA to monitor transduction efficiency. One month after injection, the mice were harvested for RT-PCR, Western blot and immunochemistry analyses. This study was carried out in strict accordance with the recommendations in the Guide for the Care and Use of Laboratory Animals of the National Institutes of Health. The protocol was approved by the Mayo Clinic Institutional Animal Care and Use Committee (Protocol number A34210). Mice were terminally anesthetized with sodium pentobarbital, and all efforts were made to minimize suffering.

### Generation of Mouse Specific Tdp-43 Antibody

The polyclonal mTdp-43 antibody is produced by using synthetic peptide corresponding to residues 203–221 of mTDP-43 (TAEELQQFFCQYGEVVDVF) to immunize rabbits. The resulting sera were affinity purified and the specificity of the antibody was characterized using Western blot.

### Quantitative Real-time PCR (qRT-PCR)

TRIzol (Invitrogen, Carlsbad, CA) and Pure Link™ RNA Mini Kit (Invitrogen, Carlsbad, CA) were used for RNA extraction. A total of 3 µg of RNA were used to synthesize cDNA using the High Capacity cDNA Reverse Transcription Kit (Applied Biosystems). The qRT- PCR was performed using an HT7900 analyzer (Applied Biosystems). Each 5 µl reaction contained: 2 µl of cDNA diluted 1∶20, 0.5 µl primer mix (1 µM for each primer), and 2.5 µl SYBR green (Invitrogen). The qRT-PCR program was as follows: 50°C 2min, 95°C 2min, 40 cycles of 95°C 15s and 60°C 1min. For dissociation curves, a dissociation stage of 95°C 15s, 60°C 15s was added at the end of the program. *Tardbp*, *mSort1+Ex17b* and *mTsn_*Δ*Ex5* mRNA values were normalized to *Gapdh* values, an endogenous transcript control. The data was analyzed by using Software RQ Manager 1.2 (Applied Biosystems). The primers used were: *Gapdh*: 5′-CATGGCCTTCCGTGTTCCTA-3′ and 5′-CCTGCTTCACCACCTTCTTGAT-3′; *Tardbp*: 5′-GGGGCAATCTGGTATATGTTG-3′ and 5′-TGGACTGCTCTTTTCACTTTCA-3′; *mSort1+Ex17b*: 5′-AAATCCCAGGAGACAAATGC-3′ and 5′-GAGCTGGATTCTGGGACAAG-3′; *mTsn_*Δ*Ex5*∶5′GGTCTTCCTGGCAGCATTTG-3′ and 5′-TTGACAGACAGCCTCGATGC-3′. To quantify the RNA levels of TDP-43 synaptic targets in human TDP-43 transgenic mice, we also performed q-PCR. There primers used were: *Psd-95*∶5′-CGCTACCAAGATGAAGACACG-3′ and 5′-CAATCACAGGGGGAGAATTG-3′; *Bsn*: 5′-TGTGGCTTTAACCCAACACC-3′ and 5′-TTTGGCAGTTCAGACAGAGC-3′; *Dlg1*∶5′-TCATTCTCATATCTCACCAATAAAGC-3′ and 5′-CAGGGGTCACAGGGACAA-3′; *Dlg2*∶5′-TCAACTCCCTACCCCCACTA-3′ and 5′-GCAGTACTGTGCTGGGAATG-3′; *Dvl1*∶5′-CCATGGACCAGGACTTCG-3′ and 5′-GGCAACTTGGCATTGTCAT-3′; *Gap-43*∶5′-AGAGGATGCTGCCACCAA-3′ and 5′-GGCTTCGTCTACAGCGTCTTT-3′; *Syt7*∶5′-GAGGCTTGGACATGAAATCC-3′ and 5′-TCCGAAAGCCCTAATACCAG-3′; *Snap25*∶5′-GCTCCTCCACTCTTGCTACC-3′ and 5′-CAGCAAGTCAGTGGTGCTTC-3′; *Syt1*∶5′-AAGGAGATTCCAAAAGGAACAA-3′ and 5′-TTTTGGTTCAAGCGGAATG-3′.

For semi-quantitative PCR, 10 µl of 1∶40 diluted cDNA was used for PCR amplification in a final volume of 20 µl using Taq polymerase (Qiagen). PCR cycling conditions were as follows: 94°C, 3min; 94°C, 30s; 60°C, 30s; 72°C, 30s (10 cycles); 94°C, 30s; 50°C, 30s; 72°C, 30s (20 cycles); 72°C, 10min. PCR products were electrophoresed on 2% agarose gels. The primers used were: *Sort1*∶5′-CAAATGCCAAGGTGGGATGAA-3′ and 5′-TTGAATCCAAAGCCTCTACGCC-3′; *Tsn*: 5′-CCCGAGAGGCTGTTACAGAG-3′ and 5′-CCTCGGATGGAAAGGTCATA-3′.

### Western Blotting

Tissues were homogenized at 10 ml/g (volume/weight) in lysis buffer (50 mM Tris-HCl, pH 7.4, 300 mM NaCl, 1% Triton X-100, 5 mM EDTA, 2% SDS, PMSF, and protease and phosphatase inhibitors). Following centrifugation, protein concentration of the supernatant was assessed by BCA assay (Pierce, Rockford, IL). Following Western blotting, membranes were incubated with rabbit polyclonal mouse specific Tdp-43 antibody that we have generated(1∶1000), mouse monoclonal human specific TDP-43 antibody (1∶1000, 2E2-D3, Novus Biologicals), rabbit polyclonal GFP antibody (1∶1000, Invitrogen), and mouse monoclonal Gapdh antibody (1∶10000, Biodesign) overnight at 4°C. Following incubation with an appropriate secondary antibody, immunoreactivity was visualized by ECL and exposure to film.

### Immunohistochemistry

Tissues were embedded in paraffin, sectioned (5 µm thick) and mounted on glass slides. Sections were deparaffinized in xylene and rehydrated in a graded series of alcohol, followed by dH_2_O. Antigen retrieval was performed in a dH_2_O steam bath for 30 min. Tissues were immunostained with cytochrome oxidase subunit IV (Cox-IV; 1∶3000; Abcam) and pS403/S404-phosphorylated TDP-43 (1∶2000; Cosmo Bio) antibodies, using the DAKO Autostainer (Dako Auto Machine Corporation) and the DAKO EnVision+ HRP system. DAKO Liquid DAB Substrate–Chromogen system was the chromogen. After immunostaining, sections were briefly counterstained with hematoxylin to visualize cell nuclei and coverslipped. Paraffin-embedded sections were also stained with hematoxylin and eosin.

### DiI-labeling

Mice were deeply anesthetized with a mixture of ketamine (100 mg/kg, i.p.) and xylazine (10 mg/kg, i.p.) and perfused with 4% paraformaldehyde (PFA). Brains were removed and put into 4% PFA for additional 20 minutes and then washed overnight in PBS at 4°C. Vibratome sections of 200 µM were prepared and immersed in PBS at 4°C. The Helios Gene Gun System (165–2431; Bio-Rad, Hercules, CA) was used to deliver gold (1.0 µm) and tungsten particles coated with lipophilic dyes into perfusion-fixed brain slices. Preparations of the DiOlistic bullets were performed according the methods described previously [36], [37]. A series of images with interval of 0.5 µm in the Z direction was taken with confocal microscope with 63×oil-immersion lens and image stacks were then generated by Image J software. The stacked images were then used to count dendritic spines by using Metamorph software. Dendritic spines of hippocampus were counted by scrolling through the z plane about 10 µm length of each segment. The number of spines in each segment was counted and the data was expressed at dendritic spines/10 µm.

### AAV1 production

AAV1-sh*Tardbp* and AAV1-shControl was prepared by standard methods. Briefly, AAV vectors expressing sh*Tardbp* and shControl under the control of the cytomegalovirus enhancer/chicken β-actin promoter, a woodchuck post-transcriptional regulatory element, and the bovine growth hormone, poly (A), were generated by plasmid transfection with helper plasmids in HEK293T cells. All plasmids were sequence verified as described above. Forty-eight hours after transfection, the cells were harvested and lysed in the presence of 0.5% sodium deoxycholate and 50 U/ml Benzonase (Sigma, St. Louis, MO) by freeze thawing, and the virus was isolated using a discontinuous iodixanol gradient. The genomic titer of each virus was determined by qRT-PCR.

### Statistics

One-way ANOVA with Tukey’s posthoc analysis were used to compare measures among 3 groups. Two-tailed student’s t test was used to compare measures among 2 groups. For data presentation, normalized values were averaged and presented as mean ± standard error of mean (SEM). Values of p<0.05 were considered statistically significant.

## Supporting Information

Figure S1 **Intraventricular injection of the neonatal mouse brain with AAV1-sh** ***Tardbp*** **can further decrease mTdp-43 levels in the brain of hTDP-43_M337V_ transgenic mice, but cannot cause loss of mTdp-43 function.** (**A–B**) Intraventricular injection of AAV1-sh*Tardbp* decreases mTdp-43 protein levels (**A–B**) and RNA levels (**C**) in the brain of both non-transgenic (NT) and hTDP-43_M337V_ hemizygous mice. There are no significant difference of the protein RNA levels of mTdp-43 between AAV1-shTDP-43 injected hemizygous mice and homozygous mice. (**D–E**) Injection of AAV1-sh*Tardbp* significantly increases the mRNA levels of *mSort1+Ex17b* (**D**) and *mTsn_* Δ*Ex5* in the brain of NT mice, but not in hTDP-43_M337V_ hemizygous mice. *mSort1+Ex17b* and *mTsn_*Δ*Ex5* RNA levels between AAV1-sh*Tardbp* injected hemizygous mice and homozygous mice are not significantly different. Data shown are the means ± SEM of 3–5 mice per group; **p<0.001, N.S. no significance, as assessed by one-way ANOVA with Tukey’s posthoc analysis.
(TIFF)

Figure S2 **No alternations of RNA of synaptic proteins and dendritic spines in hTDP-43_WT_ hemizygous mice injected with AAV1-sh** ***Tardbp*** **.** (**A**) Intraventricular injection of AAV1-sh*Tardbp* in the brain of hTDP-43_WT_ hemizygous mice does not alter the RNA levels of PSD-95, Bsn, GAP-43 and Syt7. (**B–C**) Intraventricular injection of AAV1-sh*Tardbp* in hTDP-43_WT_ hemizygous mice does not decrease dendritic spine number in the brain. Data shown are the means ± SEM of 3–5 mice per group; **p<0.001, as assessed by one-way ANOVA with Tukey’s posthoc analysis.
(TIFF)
