# Supplementary figures and images for: The Pathological Phenotypes of Human TDP-43 Transgenic Mouse Models Are Independent of Downregulation of Mouse Tdp-43
Source: PLoS One. 2013 Jul 26;8(7):e69864. doi: 10.1371/journal.pone.0069864 (PMC3724736; doi:10.1371/journal.pone.0069864)

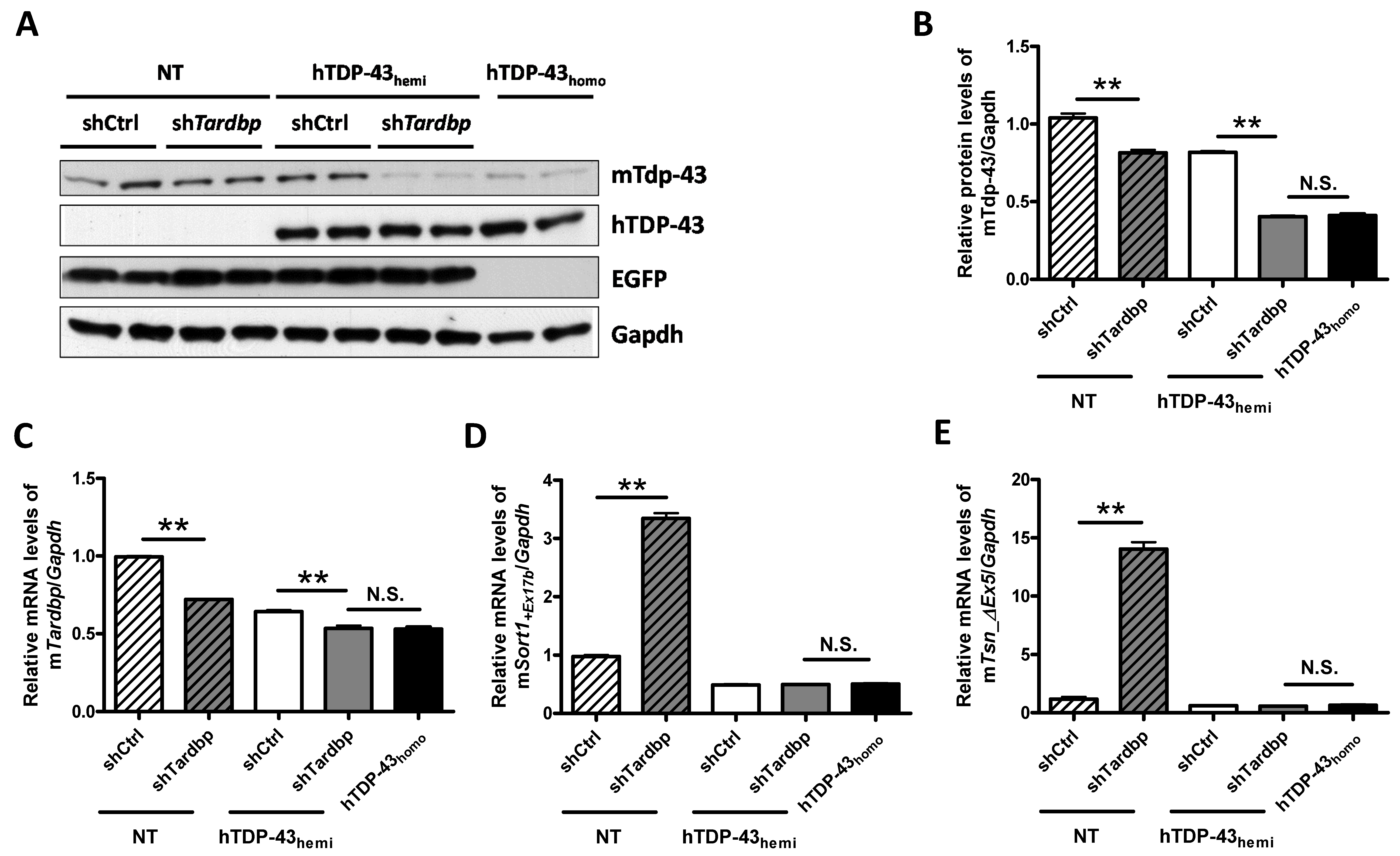

Supplement: Figure S1 — Intraventricular injection of the neonatal mouse brain with AAV1-sh Tardbp can further decrease mTdp-43 levels in the brain of hTDP-43M337V transgenic mice, but cannot cause loss of mTdp-43 function. (A–B) Intraventricular injection of AAV1-shTardbp decreases mTdp-43 protein levels (A–B) and RNA levels (C) in the brain of both non-transgenic (NT) and hTDP-43M337V hemizygous mice. There are no significant difference of the protein RNA levels of mTdp-43 between AAV1-shTDP-43 injected hemizygous mice and homozygous mice. (D–E) Injection of AAV1-shTardbp significantly increases the mRNA levels of mSort1+Ex17b (D) and mTsn_ ΔEx5 in the brain of NT mice, but not in hTDP-43M337V hemizygous mice. mSort1+Ex17b and mTsn_ΔEx5 RNA levels between AAV1-shTardbp injected hemizygous mice and homozygous mice are not significantly different. Data shown are the means ± SEM of 3–5 mice per group; **p<0.001, N.S. no significance, as assessed by one-way ANOVA with Tukey’s posthoc analysis. (TIFF) [file pone.0069864.s001.tiff]

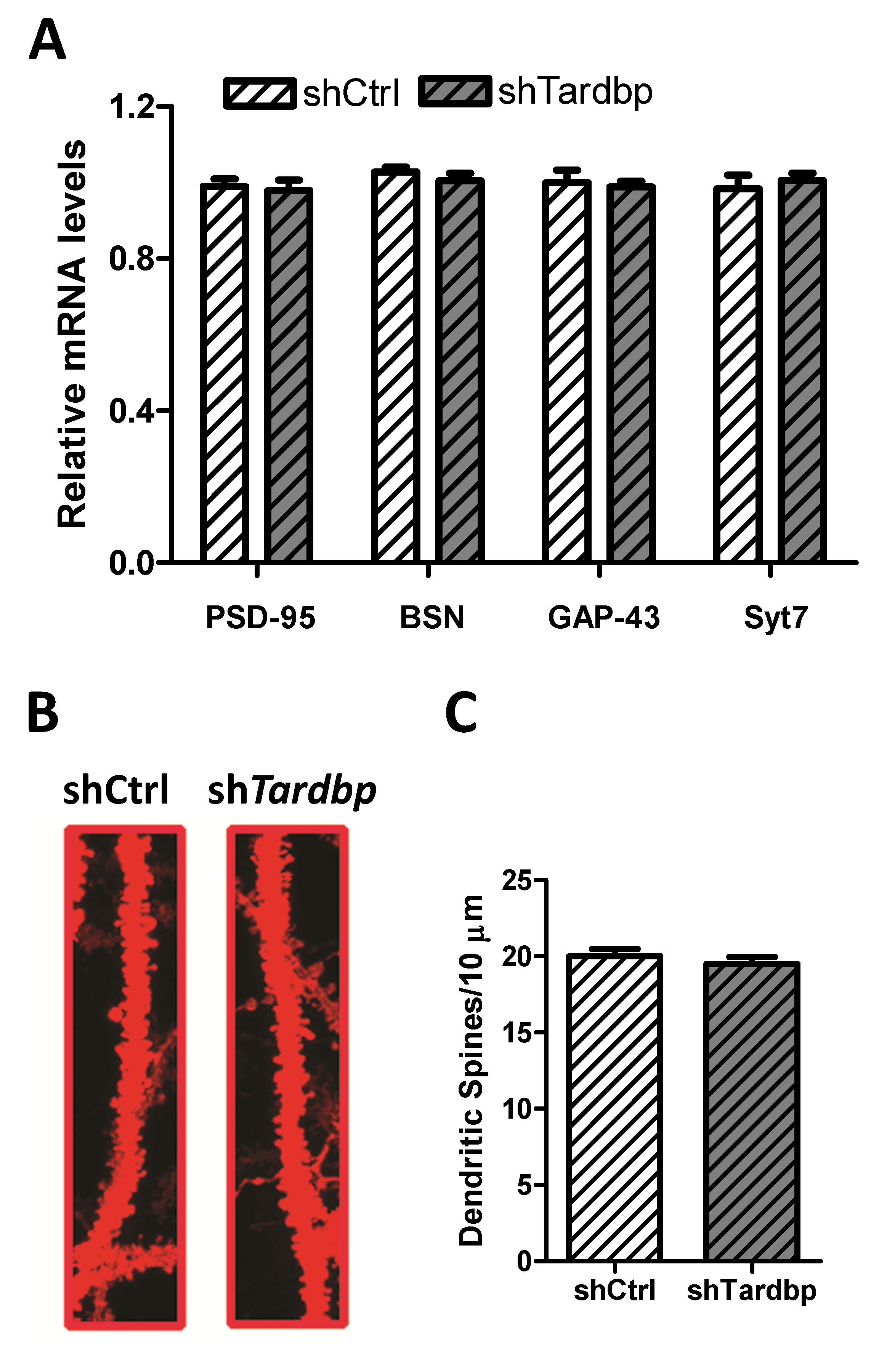

Supplement: Figure S2 — No alternations of RNA of synaptic proteins and dendritic spines in hTDP-43WT hemizygous mice injected with AAV1-sh Tardbp . (A) Intraventricular injection of AAV1-shTardbp in the brain of hTDP-43WT hemizygous mice does not alter the RNA levels of PSD-95, Bsn, GAP-43 and Syt7. (B–C) Intraventricular injection of AAV1-shTardbp in hTDP-43WT hemizygous mice does not decrease dendritic spine number in the brain. Data shown are the means ± SEM of 3–5 mice per group; **p<0.001, as assessed by one-way ANOVA with Tukey’s posthoc analysis. (TIFF) [file pone.0069864.s002.tiff]
